# Neurological manifestations following cured malaria: don't forget post-malaria neurological syndrome

**DOI:** 10.4314/ahs.v21i1.35

**Published:** 2021-03

**Authors:** Foued Bellazreg, Dorsaf Slama, Nadia Ben Lasfar, Maha Abid, Houneida Zaghouani, Sana Rouis, Wissem Hachfi, Amel Letaief

**Affiliations:** 1 Department of Infectious Diseases, Farhat Hached University Hospital, 4000 Sousse, Tunisia; 2 Department of Radiology, Farhat Hached University Hospital, 4000 Sousse, Tunisia; 3 University of Sousse, Faculté de Médecine Ibn Al Jazzar, 4000 Sousse, Tunisia

**Keywords:** Post-malaria neurological syndrome, immunologic, corticosteroid

## Abstract

**Introduction:**

Cerebral malaria which occurs during the active infection is the most common neurological complication of malaria. Other complications including post-malaria neurological syndrome (PMNS) can rarely occur following complete recovery from the disease. We report a case of post-malaria neurological syndrome in a Tunisian patient.

**Case presentation:**

A 26-year-old Tunisian man with no past medical history was admitted in 2016 for a muscle weakness of the 4 limbs, seizures, tetraparesis and myoclonus which appeared after he returned from Côte d'Ivoire where he had been treated three weeks ago for *Plasmodium falciparum* malaria with favorable outcome. Blood smears for malaria were negative. Brain MRI showed multiple hypersignal cerebral lesions. Investigations didn't show any infectious, metabolic, toxic, vascular or tumoral etiology. Thus, the diagnosis of PMNS was considered. The patient was treated with methylprednisolone with favorable outcome. Two years later, he was completely asymptomatic.

**Conclusion:**

PMNS should be considered in patients with neurological symptoms occurring within two months of cured acute disease in which blood smears for malaria are negative and other etiologies have been ruled out. In most cases, the disease is self-limited while in severe cases corticosteroid therapy should be prescribed with favorable outcome.

## Introduction

In 2018, the World Health Organization (WHO) estimated that 228 million cases of malaria occurred worldwide and caused 405 000 deaths. *Plasmodium falciparum*, the most prevalent malaria parasite, is most commonly associated with severe disease namely neurological complications and mortality 1. Among the neurological complications, cerebral malaria is by far the most common. It occurs during the active infection and can lead to death or to residual neurological sequelae in 15–20% and 1–3% of treated patients respectively 2. Other neurological complications can rarely occur following complete recovery from malaria. They include post-malaria neurological syndrome (PMNS), acute disseminated encephalomyelitis, delayed cerebellar ataxia and acute inflammatory demyelinating polyneuropathy^3^. In Tunisia, malaria has been eradicated since 1979 but imported cases from travelers returning from endemic countries continue to be observed. We report here a case of PMNS in a Tunisian patient after his return from Côte d'Ivoire.

## Case presentation

A 26-year-old Tunisian man with no past medical history, who worked in Côte d'Ivoire since September 2015, was admitted in July 2016 for a 48-hour history of tetraparesis tonic-clonic generalized seizures and myoclonus. These signs appeared after he returned from Côte d'Ivoire where he had been treated three weeks ago for *Plasmodium falciparum* malaria with favorable outcome.

**Figure 1:**
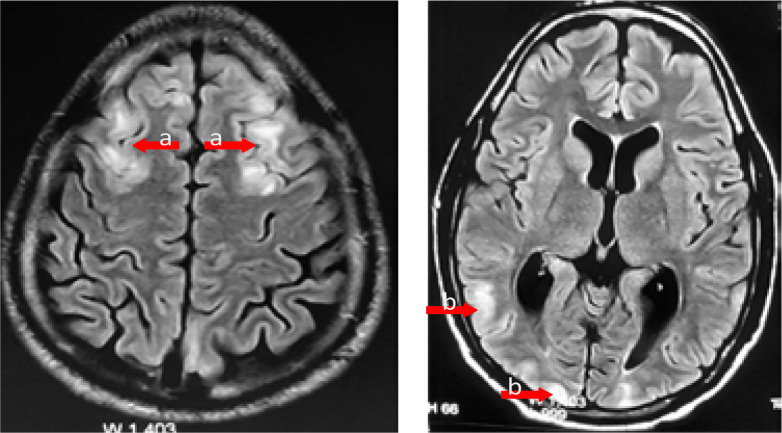
Initial T2-flair MRI showing hypersignal cerebral lesions (arrows) predominantly in the white matter of the frontal (a) and the occipital lobes (b).

Medical history didn't reveal any alcohol or illicit drugs consumption. Physical examination showed tetraparesis and was unremarkable otherwise, namely there were no dysarthria, cranial nerve palsies, ataxia, pyramidal signs or sensory deficits. Laboratory data including blood sugar, sodium, calcium, creatinine, blood cell count, prothrombin time and cerebrospinal fluid (CSF) analysis were normal, and blood toxicologic analysis was negative. Peripheral blood smears were both negative for *Plasmodium*. Brain CT scan was normal but brain and spine magnetic resonance imaging (MRI) showed multiple T2-flair hypersignal cerebral lesions predominantly in the white matter of the frontal and the occipital lobes (Figure 1) without myelitis or radiculitis.

The diagnosis of infectious encephalitis was initially considered and the patient was treated with intravenous aciclovir associated to anticonvulsant therapy. Serologic tests for West Nile virus, human immunodeficiency virus, cytomegalovirus, Ebstein-Barr virus, hepatitis B, hepatitis C, *Brucella, Rickettsia* and *Coxiella burnetii* were all negative, and echocardiography was normal.

**Figure 2:**
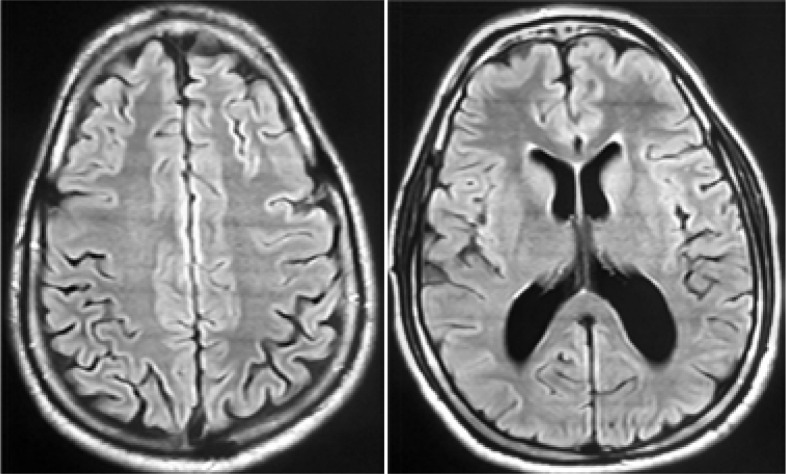
End-of-therapy MRI, without abnormalities

At day 5 of aciclovir, the patient has not improved and PCR for HSV1 and HSV2 in the CSF was negative. Aciclovir was then stopped and the diagnosis of PMNS was considered. The patient was treated with oral methylprednisolone 1 mg/kg daily with progressive improvement of the neurological deficits. At day 15 of methylprednisolone he was discharged. Six weeks later, he had no neurological deficit or other complaints and brain MRI was without abnormalities (Figure 2). The corticosteroid therapy was discontinued after a total duration of 8 weeks and the patient returned to his job in Côte d'Ivoire. Two years later, he was completely asymptomatic.

## Discussion

Among the four neurological complications reported after full recovery of malaria, our patient had no signs of delayed cerebellar ataxia, acute inflammatory demyelinating polyneuropathy or acute disseminated encephalomyelitis. Thus, he was considered to have PMNS. PMNS, first reported in 1996 by Nguyen et al. in 22 Vietnamese patients, was defined by the occurrence of neurological or psychiatric symptoms after a symptom-free period within two months following cured acute malaria with a negative blood smear and no alternative diagnosis 4. It is a rare condition with an incidence of 1.2–1.7 per 1000 malaria cases 4,5. Until February 2020, only 54 cases of PMNS were reported including 28 of them were isolated case reports 3,5,6. In two recent systematic reviews, most cases of PMNS were observed in young adult males (mean age, 33 years) and preceded by severe *Plasmodium falciparum* malaria. They occurred within a mean duration of 9 days (0–60) after the malaria recovery. A wide range of clinical signs were reported including impaired consciousness, fever, seizures, motor deficits, myoclonus, tremor and ataxia 3,5. CSF analysis showed lymphocytic meningitis in 60% of cases and high protein levels in 77% of cases while glucose level was normal in all patients 5. Among imaging techniques, CT scan was normal in all cases, while MRI revealed brain abnormalities in 43% of cases. These abnormalities consisted of nonspecific hypersignals sometimes associated with gadolinium enhancement in different localizations including sub-cortical and periventricular areas, iternal capsule, corpus callosum, thalamus, brain stem and cerebellum 3,5.

Based on clinical severity, Schnorf et al. classified the syndrome into three categories: a mild form with isolated cerebellar ataxia or postural tremor, a diffuse mild encephalopathy with confusion or seizures, and a severe encephalopathy with motor aphasia, generalized myoclonus, postural tremor and cerebellar ataxia 7.

In our patient, neurological manifestations which consisted of seizures, tetraparesis and myoclonus appeared 3 weeks after full recovery of *Plasmodium falciparum* malaria. CSF and CT scan were normal while MRI showed multiple T2-flair hypersignal cerebral lesions predominantly in the white matter of the frontal and the occipital lobes. Peripheral blood smears were negative for *Plasmodium* and infectious, metabolic, toxic, vascular and tumoral etiologies were ruled out by laboratory and imaging investigations. Based on these findings, the diagnosis of PMNS in its mild encephalopathy form with seizures has been established.

The pathogenesis of PMNS is not well understood. The delayed-onset after a 2–3 weeks symptom-free period the negativity of blood smears, the high CSF concentrations of pro-inflammatory cytokines and the beneficial effect of corticosteroid therapy are highly suggestive of the immunological mechanism of this disease 3,5,7,8. The cerebral microvasculature where parasites are sequestered due to cytoadherence could be the locus of immunization process. The cytoadherence is less frequent and less significant in *Plasmodium vivax* infection which could explain why most PMNS cases follow *Plasmodium falciparum* infections 9,10.

PMNS is usually self-limited and does not require specific treatment. However, in severe cases which occur in almost 25% of patients, corticosteroids have been used to hasten recovery 3,7. In our patient, based on MRI data which showed multiple brain lesions and on the absence of spontaneous improvement, corticosteroid therapy with methylprednisolone was prescribed during 8 weeks and the outcome was favorable.

## Conclusion

PMNS should be considered in patients with neurological symptoms occurring within two months of cured acute disease in which blood smears for malaria are negative and other etiologies have been ruled out. In most cases, the disease is self-limited while in severe cases corticosteroid therapy should be prescribed with favorable outcome.
